# Crystal structure of di­chlorido­(1,2-phenyl­enedi­amine-κ^2^ *N*,*N*′)platinum(II)

**DOI:** 10.1107/S2056989017008477

**Published:** 2017-06-13

**Authors:** Yosuke Konno, Nobuyuki Matsushita

**Affiliations:** a Department of Chemistry, Graduate School of Arts and Sciences, The University of Tokyo, Komaba 3-8-1, Meguro-ku, Tokyo, 153-8902, Japan; b Department of Chemistry & Research Center for Smart Molecules, Rikkyo University, Nishi-Ikebukuro 3-34-1, Toshima-ku, Tokyo, 171-8501, Japan

**Keywords:** crystal structure, columnar structure, infinite metal chain, platinum(II) complex, hydrogen bonding

## Abstract

The Pt^II^ atom in the title compound, [PtCl_2_{(C_6_H_4_)(NH_2_)_2_}], lies on a twofold rotation axis and has a slightly distorted square-planar coordination environment defined by two N atoms of an 1,2-phenyl­enedi­amine ligand and two Cl^−^ ions. In the crystal, the planar complex mol­ecules are stacked parallel to the *c* axis, resulting in a columnar structure. In a column, an infinite almost straight Pt⋯Pt chain is formed, suggesting weak metal–metal inter­actions [Pt⋯Pt = 3.3475 (8) Å]. The crystal packing is stabilized by a three-dimensional N—H⋯Cl hydrogen-bonding network between the amino groups and the Cl ligands of adjacent mol­ecules.

## Abstract 2

In the crystal structure of the title compound, almost planar [PtCl_2_{(C_6_H_4_)(NH_2_)_2_}] mol­ecules are stacked into columns along the *c* axis, suggesting Pt⋯Pt inter­actions.

## Chemical context   

The title compound, di­chlorido­(1,2-phenyl­enedi­amine-κ^2^ *N*,*N*′)platinum(II) [PtCl_2_{(C_6_H_4_)(NH_2_)_2_}], (I), which was originally prepared by Connors *et al.* (1972 ▸), is a member of the family of derivatives of *cis*-diamminedi­chlorido­platinum(II), *cis*-[PtCl_2_(NH_3_)_2_] (*cis*-platin). Since the discovery of the anti­tumor activity of *cis*-platin (Rosenberg *et al.*, 1965 ▸), numerous derivatives and analogues of *cis*-platin have been prepared and investigated. However, reports on the corresponding crystal structures are rather scarce, probably because of the difficulty in obtaining crystals suitable for X-ray analysis, in part owing to poor solubility. Although the anti­tumor activity (Connors *et al.*, 1972 ▸; Meischen *et al.*, 1976 ▸) and the chemical stabilities (Köckerbauer & Bednarski, 1996 ▸) of the title compound have been reported, its crystal structure has not been determined so far. In the course of our study of the deprotonation and redox properties of a platinum complex with 1,2-phenyl­enedi­amine as a ligand (Konno & Matsushita, 2006*a* ▸,*b* ▸), we have successfully obtained single crystals of the title compound and report here its crystal structure.
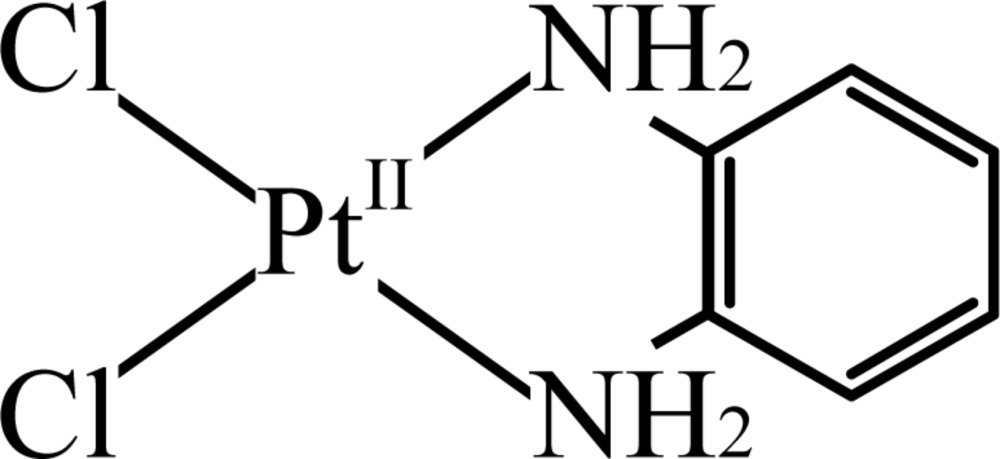


## Structural commentary   

**Figure 1:**
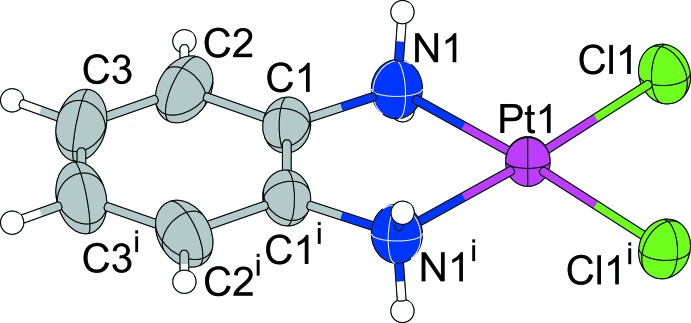
A view of the mol­ecular structure of compound (I), showing the atomic numbering scheme. Displacement ellipsoids are drawn at the 50% probability level for non-H atoms. [Symmetry code: (i) −*x* + 1, *y*, −*z* + 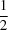.]

The mol­ecular structure of (I) is displayed in Fig. 1 ▸. The platinum compound (I) is isostructural with the palladium compound [PdCl_2_{(C_6_H_4_)(NH_2_)_2_}] reported previously (Konno & Matsushita, 2017 ▸). The Pt^II^ atom lies on a twofold rotation axis. Hence the asymmetric unit comprises half of a [PtCl_2_{(C_6_H_4_)(NH_2_)_2_}] mol­ecule, the other half being completed by application of twofold rotation symmetry. The Pt^II^ atom is coordinated by two N atoms of an 1,2-phenyl­enedi­amine ligand and by two Cl^−^ ions in a slightly distorted square-planar configuration (Table 1 ▸). The r.m.s. deviation of the least-squares plane formed by atoms Pt1, N1, C1, C2 and C3 is 0.0121 Å. The structural parameters of the coordination sphere around Pt^II^ in the crystal of (I) (Table 1 ▸) are consistent with those found in *cis*-[PtCl_2_(NH_3_)_2_] (Milburn & Truter, 1966 ▸), [PtCl_2_(en)] (en is ethyl­enedi­amine; Iball *et al.*, 1975 ▸), *cis*-[PtCl_2_(*L*)_2_] (*L* is cyclo­hexyl­amine; Lock *et al.*, 1980 ▸), [PtCl_2_(*cis*-dac)]·0.33-hydrate (dac is 1,2-di­amino­cyclo­hexane; Lock & Pilon, 1981 ▸), *cis*-[PtCl_2_(*L′*)(NH_3_)] (*L′* is cyclo­butyl­amine; Rochon & Melanson, 1986 ▸), [PtCl_2_(Me_2_en)] (Me_2_en is *N*,*N*-di­methyl­ethylenedi­amine; Melanson *et al.*, 1987 ▸), [PtCl_2_(tn)] (tn is 1,3-di­amino­propane; Odoko & Okabe, 2006 ▸), [PtCl_2_(*L′′*)] (*L′′* is 2-morpholino­ethyl­amine; Shi *et al.*, 2006 ▸), [PtCl_2_(Me_4_en)] (Me_4_en is *N*,*N*,*N′*,*N′*- tetra­methyl­ethylenedi­amine; Asiri *et al.*, 2012 ▸). Bond lengths and angles of the 1,2-phenyl­enedi­amine moiety (Table 1 ▸) are not significantly different from those found in the bis­(1,2-phenyl­enedi­amine)­platinum(II) complex, [Pt(C_6_H_8_N_2_)_2_]Cl_2_·2H_2_O [N—C = 1.450 (2) Å, C—C = 1.365 (6)–1.389 (4) Å; Konno & Matsushita, 2006*a* ▸] or in isostructural di­chlorido­(1,2-phenyl­enedi­amine)­palladium(II) [N—C = 1.458 (2) Å, C—C = 1.371 (3)–1.416 (8) Å; Konno & Matsushita, 2017 ▸].

## Supra­molecular features   

**Figure 2:**
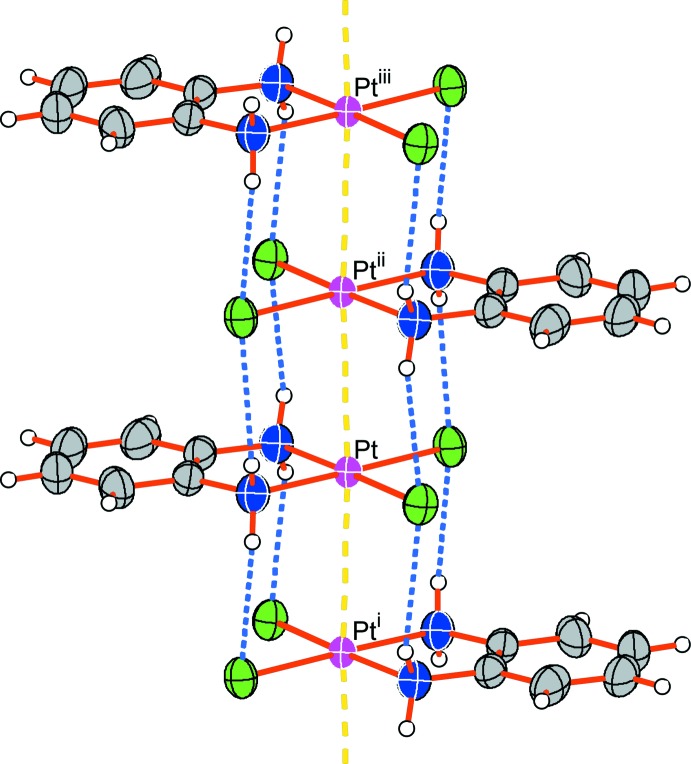
A view of the columnar structure of compound (I). Light-blue dashed lines represent hydrogen bonds between adjacent mol­ecules in the column. Yellow dashed lines indicate the short contact between Pt atoms in the column. [Symmetry codes: (i) −*x* + 1, −*y* + 1, −*z*; (ii) −*x* + 1, −*y* + 1, −*z* + 1; (iii) *x*, *y*, *z* + 1.]

As shown in Fig. 2 ▸, the neutral planar mol­ecules of (I) stack parallel to the *c* axis, resulting in a columnar structure. The planar [PtCl_2_{(C_6_H_4_)(NH_2_)_2_}] units are arranged in parallel and the 1,2-phenyl­enedi­amine moieties alternate with each other as a result of the *c*-glide operation. In the column, an infinite, almost straight [Pt⋯Pt⋯Pt = 176.513 (11)°] platinum chain is formed with a short inter­atomic distance [Pt⋯Pt = 3.3475 (8) Å], suggesting weak metal–metal inter­actions. The infinite palladium chain of the isostructural Pd complex is straighter [Pd⋯Pd⋯Pd = 179.232 (7)°] than the platinum chain. The Pt⋯Pt distance in (I) is slightly shorter than those of *cis*-[PtCl_2_(NH_3_)_2_] [3.372 (2) and 3.409 (2) Å; Milburn & Truter, 1966 ▸] or [PtCl_2_(en)] [3.381 Å; Iball *et al.*, 1975 ▸], and is considerably shorter than that of [PtCl_2_(tn)] [3.646 Å; Odoko & Okabe, 2006 ▸], all of which have similar columnar structures.

**Figure 3:**
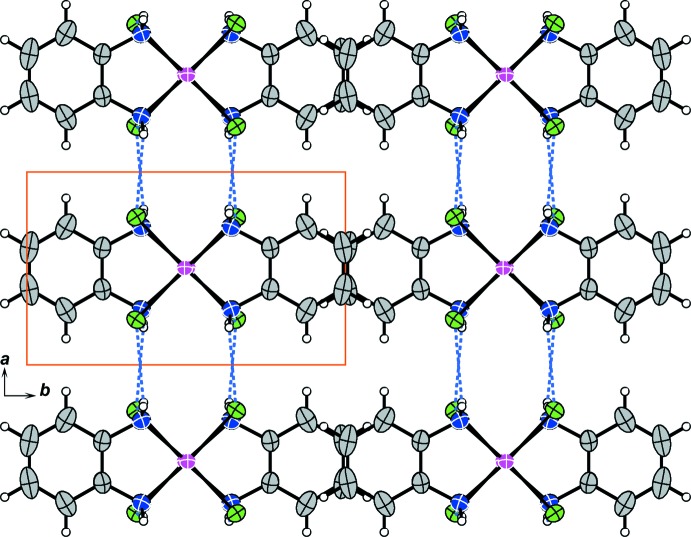
The crystal packing of compound (I), viewed along the *c* axis. Light-blue dashed lines represent inter­columnar hydrogen bonds. Solid orange lines indicate the unit cell.

The inter­molecular Pt⋯Pt distance of (I) suggests that the columnar structure is stabilized by weak metal–metal inter­actions. The columnar structure of (I) is further stabilized by inter­molecular N—H⋯Cl hydrogen bonds between adjacent mol­ecules in the column (Fig. 2 ▸ and Table 2 ▸). Inter­columnar hydrogen bonds also help to stabilize the crystal packing of the columns (Fig. 3 ▸, and Table 2 ▸).

## Synthesis and crystallization   

Compound (I) was prepared using a method modified from that described by Connors *et al.* (1972 ▸) as follows. To an aqueous HCl solution (1.0 *M*, 15 ml) of K_2_[PtCl_4_] (0.241 mmol, 100 mg) was slowly added an aqueous HCl solution (1.0 *M*, 15 ml) of 1,2-phenyl­enedi­amine (0.241 mmol, 26 mg), and then the solution was sealed in a screw-cap vial and was kept at room temperature for one week in the dark. Pale-brown needle-like crystals suitable for X-ray analysis were obtained (yield 52%). Elemental analysis found: C 19.26, H 2.23, N 7.30%; calculated for C_6_H_8_Cl_2_N_2_Pt: C 19.26, H 2.16, N 7.49%. Elemental analysis was carried out by the Laboratory of Organic Elemental Analysis, Department of Chemistry, Graduate School of Science, The University of Tokyo.

## Refinement   

Crystal data, data collection and structure refinement details are summarized in Table 3 ▸. One reflection (010) was omitted in the final refinement because it was obstructed by the beam-stop. H atoms were placed in geometrically calculated positions and refined as riding, with C(aromatic)—H = 0.93 and N—H = 0.90 Å, and with *U*_iso_(H) = 1.2*U*_eq_(C,N). The maximum and minimum electron density peaks are located 0.80 and 0.74 Å, respectively, from atom Pt1.

## Supplementary Material

Crystal structure: contains datablock(s) global, I. DOI: 10.1107/S2056989017008477/wm5396sup1.cif

Structure factors: contains datablock(s) I. DOI: 10.1107/S2056989017008477/wm5396Isup2.hkl

CCDC reference: 1055177

Additional supporting information:  crystallographic information; 3D view; checkCIF report

## Figures and Tables

**Table 1:** Selected geometric parameters (Å, °)

Pt1—N1	2.040 (4)	C1—C2	1.372 (6)
Pt1—Cl1	2.3213 (13)	C1—C1^ii^	1.386 (9)
Pt1—Pt1^i^	3.3475 (8)	C2—C3	1.377 (11)
N1—C1	1.445 (6)	C3—C3^ii^	1.38 (3)
			
N1—Pt1—N1^ii^	83.6 (3)	Pt1^i^—Pt1—Pt1^iii^	176.513 (11)
N1—Pt1—Cl1	91.39 (15)	C1—N1—Pt1	110.8 (3)
Cl1^ii^—Pt1—Cl1	93.69 (7)	C2—C1—C1^ii^	119.8 (3)
N1—Pt1—Pt1^i^	92.07 (14)	C2—C1—N1	122.7 (5)
Cl1—Pt1—Pt1^i^	93.80 (4)	C1^ii^—C1—N1	117.4 (2)
N1—Pt1—Pt1^iii^	85.32 (14)	C1—C2—C3	120.3 (8)
Cl1—Pt1—Pt1^iii^	88.59 (4)	C2—C3—C3^ii^	119.8 (6)

Symmetry codes: (i) 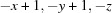; (ii) 
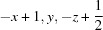
; (iii) 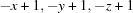.

**Table 2:** Hydrogen-bond geometry (Å, °)

*D*—H⋯*A*	*D*—H	H⋯*A*	*D*⋯*A*	*D*—H⋯*A*
N1—H1*A*⋯Cl1^iv^	0.90	2.57	3.353 (4)	146
N1—H1*B*⋯Cl1^v^	0.90	2.71	3.381 (4)	133
N1—H1*B*⋯Cl1^vi^	0.90	2.73	3.320 (5)	124

Symmetry codes: (iv) 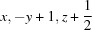; (v) 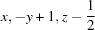; (vi) 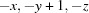.

**Table 3:** Experimental details

Crystal data	
Chemical formula	[PtCl_2_(C_6_H_8_N_2_)]
*M*_r_	374.13
Crystal system, space group	Monoclinic, *P*2/*c*
Temperature (K)	296
*a*, *b*, *c* (Å)	7.087 (2), 10.446 (3), 6.6920 (16)
β (°)	116.61 (2)
*V* (Å^3^)	442.9 (2)
*Z*	2
Radiation type	Mo *K*α
μ (mm^−1^)	16.38
Crystal size (mm)	0.26 × 0.13 × 0.07

Data collection	
Diffractometer	Rigaku R-AXIS RAPID imaging-plate
Absorption correction	Multi-scan (*ABSCOR*; Higashi, 1995 ▸)
*T*_min_, *T*_max_	0.116, 0.304
No. of measured, independent and observed [*F* ^2^ > 2σ(*F* ^2^)] reflections	10875, 1587, 1480
*R*_int_	0.030
(sin θ/λ)_max_ (Å^−1^)	0.757

Refinement	
*R*[*F* ^2^ > 2σ(*F* ^2^)], *wR*(*F* ^2^), *S*	0.034, 0.099, 1.18
No. of reflections	1587
No. of parameters	52
H-atom treatment	H-atom parameters constrained
Δρ_max_, Δρ_min_ (e Å^−3^)	4.62, −1.74

Computer programs: *RAPID-AUTO* (Rigaku, 1998 ▸), *SIR92* (Altomare *et al.*, 1994 ▸), *DIAMOND* (Brandenburg, 2017 ▸), *SHELXL97* (Sheldrick, 2008 ▸) and *publCIF* (Westrip, 2010 ▸).

## supplementary crystallographic information

### Crystal data

**Table:**

[PtCl_2_(C_6_H_8_N_2_)]	*F*(000) = 340
*M**_r_* = 374.13	*D*_x_ = 2.805 Mg m^−^^3^
Monoclinic, *P*2/*c*	Mo *K*α radiation, λ = 0.71075 Å
Hall symbol: -P 2yc	Cell parameters from 12924 reflections
*a* = 7.087 (2) Å	θ = 2.0–32.6°
*b* = 10.446 (3) Å	µ = 16.38 mm^−^^1^
*c* = 6.6920 (16) Å	*T* = 296 K
β = 116.61 (2)°	Needle, pale brown
*V* = 442.9 (2) Å^3^	0.26 × 0.13 × 0.07 mm
*Z* = 2	

### Data collection

**Table:**

Rigaku R-AXIS RAPID imaging-plate diffractometer	1587 independent reflections
Radiation source: X-ray sealed tube	1480 reflections with *F*^2^ > 2σ(*F*^2^)
Graphite monochromator	*R*_int_ = 0.030
Detector resolution: 10.00 pixels mm^-1^	θ_max_ = 32.6°, θ_min_ = 3.2°
ω scans	*h* = −10→10
Absorption correction: multi-scan (*ABSCOR*; Higashi, 1995)	*k* = −15→15
*T*_min_ = 0.116, *T*_max_ = 0.304	*l* = −10→8
10875 measured reflections	

### Refinement

**Table:**

Refinement on *F*^2^	Secondary atom site location: difference Fourier map
Least-squares matrix: full	Hydrogen site location: inferred from neighbouring sites
*R*[*F*^2^ > 2σ(*F*^2^)] = 0.034	H-atom parameters constrained
*wR*(*F*^2^) = 0.099	*w* = 1/[σ^2^(*F*_o_^2^) + (0.0669*P*)^2^ + 0.3105*P*] where *P* = (*F*_o_^2^ + 2*F*_c_^2^)/3
*S* = 1.18	(Δ/σ)_max_ = 0.001
1587 reflections	Δρ_max_ = 4.62 e Å^−^^3^
52 parameters	Δρ_min_ = −1.74 e Å^−^^3^
0 restraints	Extinction correction: SHELXL97 (Sheldrick, 2008), Fc^*^=kFc[1+0.001xFc^2^λ^3^/sin(2θ)]^-1/4^
Primary atom site location: structure-invariant direct methods	Extinction coefficient: 0.0039 (12)

### Special details

**Table:**

Geometry. All esds (except the esd in the dihedral angle between two l.s. planes) are estimated using the full covariance matrix. The cell esds are taken into account individually in the estimation of esds in distances, angles and torsion angles; correlations between esds in cell parameters are only used when they are defined by crystal symmetry. An approximate (isotropic) treatment of cell esds is used for estimating esds involving l.s. planes. Least-squares planes (x,y,z in crystal coordinates) and deviations from them (* indicates atom used to define plane) - 2.7022 (0.0123) x - 0.0000 (0.0000) y + 6.6740 (0.0026) z = 0.3174 (0.0059) * 0.0000 (0.0000) Pt1 * -0.0185 (0.0028) Cl1 * 0.0206 (0.0042) N1 * 0.0050 (0.0039) C1 * -0.0017 (0.0044) C2 * 0.0031 (0.0116) C3 * 0.0185 (0.0028) Cl1_$6 * -0.0206 (0.0042) N1_$6 * -0.0050 (0.0039) C1_$6 * 0.0017 (0.0044) C2_$6 * -0.0031 (0.0116) C3_$6 Rms deviation of fitted atoms = 0.0121
Refinement. Refinement of F^2^ against ALL reflections. The weighted R-factor wR and goodness of fit S are based on F^2^, conventional R-factors R are based on F, with F set to zero for negative F^2^. The threshold expression of F^2^ > 2sigma(F^2^) is used only for calculating R-factors(gt) etc. and is not relevant to the choice of reflections for refinement. R-factors based on F^2^ are statistically about twice as large as those based on F, and R- factors based on ALL data will be even larger.

### Fractional atomic coordinates and isotropic or equivalent isotropic displacement parameters (Å^2^)

**Table:**

	*x*	*y*	*z*	*U*_iso_*/*U*_eq_	
Pt1	0.5000	0.504875 (16)	0.2500	0.03029 (13)	
Cl1	0.23327 (19)	0.65687 (13)	0.1392 (2)	0.0429 (3)	
N1	0.2863 (7)	0.3592 (4)	0.1666 (8)	0.0407 (9)	
H1A	0.2144	0.3653	0.2480	0.049*	
H1B	0.1934	0.3657	0.0213	0.049*	
C1	0.3910 (7)	0.2364 (4)	0.2066 (6)	0.0400 (8)	
C2	0.2835 (10)	0.1225 (5)	0.1621 (9)	0.0563 (12)	
H2	0.1370	0.1224	0.1016	0.068*	
C3	0.391 (3)	0.0081 (5)	0.207 (2)	0.068 (3)	
H3	0.3182	−0.0690	0.1780	0.082*	

### Atomic displacement parameters (Å^2^)

**Table:**

	*U*^11^	*U*^22^	*U*^33^	*U*^12^	*U*^13^	*U*^23^
Pt1	0.02635 (17)	0.02835 (16)	0.03208 (18)	0.000	0.00944 (11)	0.000
Cl1	0.0335 (5)	0.0383 (5)	0.0513 (6)	0.0058 (4)	0.0140 (4)	0.0012 (4)
N1	0.0347 (18)	0.0371 (18)	0.043 (2)	−0.0017 (14)	0.0108 (16)	−0.0011 (15)
C1	0.050 (2)	0.0328 (18)	0.0365 (18)	−0.0028 (15)	0.0185 (17)	−0.0012 (14)
C2	0.067 (3)	0.046 (3)	0.054 (3)	−0.018 (2)	0.026 (2)	−0.005 (2)
C3	0.112 (10)	0.037 (3)	0.063 (6)	−0.017 (3)	0.047 (6)	−0.006 (2)

### Geometric parameters (Å, º)

**Table:**

Pt1—N1	2.040 (4)	N1—H1B	0.9000
Pt1—N1^i^	2.040 (4)	C1—C2	1.372 (6)
Pt1—Cl1^i^	2.3213 (13)	C1—C1^i^	1.386 (9)
Pt1—Cl1	2.3213 (13)	C2—C3	1.377 (11)
Pt1—Pt1^ii^	3.3475 (8)	C2—H2	0.9300
Pt1—Pt1^iii^	3.3475 (8)	C3—C3^i^	1.38 (3)
N1—C1	1.445 (6)	C3—H3	0.9300
N1—H1A	0.9000		
			
N1—Pt1—N1^i^	83.6 (3)	C1—N1—Pt1	110.8 (3)
N1—Pt1—Cl1^i^	174.82 (12)	C1—N1—H1A	109.5
N1^i^—Pt1—Cl1^i^	91.39 (15)	Pt1—N1—H1A	109.5
N1—Pt1—Cl1	91.39 (15)	C1—N1—H1B	109.5
N1^i^—Pt1—Cl1	174.82 (12)	Pt1—N1—H1B	109.5
Cl1^i^—Pt1—Cl1	93.69 (7)	H1A—N1—H1B	108.1
N1—Pt1—Pt1^ii^	92.07 (14)	C2—C1—C1^i^	119.8 (3)
N1^i^—Pt1—Pt1^ii^	85.32 (14)	C2—C1—N1	122.7 (5)
Cl1^i^—Pt1—Pt1^ii^	88.59 (4)	C1^i^—C1—N1	117.4 (2)
Cl1—Pt1—Pt1^ii^	93.80 (4)	C1—C2—C3	120.3 (8)
N1—Pt1—Pt1^iii^	85.32 (14)	C1—C2—H2	119.8
N1^i^—Pt1—Pt1^iii^	92.07 (14)	C3—C2—H2	119.8
Cl1^i^—Pt1—Pt1^iii^	93.80 (4)	C2—C3—C3^i^	119.8 (6)
Cl1—Pt1—Pt1^iii^	88.59 (4)	C2—C3—H3	120.1
Pt1^ii^—Pt1—Pt1^iii^	176.513 (11)	C3^i^—C3—H3	120.1
			
Pt1^ii^—Pt1—N1—C1	84.8 (3)	Pt1^iii^—Pt1—N1—C1	−92.9 (3)

Symmetry codes: (i) −*x*+1, *y*, −*z*+1/2; (ii) −*x*+1, −*y*+1, −*z*; (iii) −*x*+1, −*y*+1, −*z*+1.

### Hydrogen-bond geometry (Å, º)

**Table:**

*D*—H···*A*	*D*—H	H···*A*	*D*···*A*	*D*—H···*A*
N1—H1*A*···Cl1^iv^	0.90	2.57	3.353 (4)	146
N1—H1*B*···Cl1^v^	0.90	2.71	3.381 (4)	133
N1—H1*B*···Cl1^vi^	0.90	2.73	3.320 (5)	124

Symmetry codes: (iv) *x*, −*y*+1, *z*+1/2; (v) *x*, −*y*+1, *z*−1/2; (vi) −*x*, −*y*+1, −*z*.
